# Sleep Characteristics and Mental Health Among Kuwait University Students: Sensitivity Analyses for Item Overlap

**DOI:** 10.3390/bs16081446

**Published:** 2026-08-21

**Authors:** Fatima Al-Ghadban, Ahmad Salman

**Affiliations:** Department of Public Health Practice, College of Public Health, Health Sciences Center, Kuwait University, Safat 13110, Kuwait; ahmad.salman@ku.edu.kw

**Keywords:** sleep quality, sleep duration, sleep disturbance, depressive symptoms, psychological well-being, PHQ-9, WHO-5, university students, Kuwait, cross-sectional study

## Abstract

Background and Objectives: Sleep is a potentially modifiable correlate of student mental health, but associations may be inflated by content overlap between sleep and mental health measures. We examined four self-reported sleep characteristics in relation to depressive symptoms and well-being, with sensitivity analyses reducing item overlap. Materials and Methods: In this cross-sectional study, questionnaire data were collected from 389 Kuwait University students recruited by convenience from students who were concurrently participating in a campus-based physical-assessment study. Depressive symptoms used a nine-item PHQ-9-based measure (items 3, 9 modified); well-being used five WHO-5 items with modified frequency anchors. Associations used multivariable linear regression with sequential covariate adjustment. Results: For PHQ-9-based scores, independent associations were found for better sleep quality (B = −1.22, 95% CI −1.84 to −0.60; β = −0.20), more frequent disturbance (1.11, 0.49 to 1.73; β = 0.18), longer sleep duration (−0.48, −0.74 to −0.21; β = −0.17), and longer latency (0.54, 0.08 to 0.99; β = 0.11). Quality (4.01, 1.72 to 6.30; β = 0.19) and duration (1.35, 0.37 to 2.33; β = 0.14) were associated with well-being. No statistically significant sleep-by-sex interactions were detected; latency was not robust to item-3 removal. Conclusions: These findings support considering sleep health within university mental health initiatives; longitudinal studies are needed to establish directionality and intervention effects.

## 1. Introduction

Mental health concerns among university students are a public health priority for prevention and early identification. The transition to university is often accompanied by increased academic demands, changing social roles, financial pressures, and disruptions in health-related behaviors. International surveys consistently report a substantial burden of common mental disorders in student populations (Auerbach et al., 2018). Within this context, sleep represents a potentially modifiable health behavior relevant to both negative mental health outcomes, such as depressive symptoms, and positive outcomes, including psychological well-being.

Sleep health is a multidimensional construct. In addition to sleep duration, it encompasses subjective quality, continuity, timing, regularity, alertness, and efficiency (Buysse, 2014). Current adult recommendations suggest at least 7 h of sleep per night on a regular basis; however, adequate duration does not necessarily indicate restorative or high-quality sleep (Hirshkowitz et al., 2015; Watson et al., 2015). Sleep-onset latency, recurrent sleep disturbances, and subjective sleep quality therefore provide complementary information not captured by duration alone (Ohayon et al., 2017). University students are particularly vulnerable to insufficient and irregular sleep due to academic workload, social commitments, technology use, employment, and circadian misalignment that may conflict with institutional timetables and other daily demands (Hershner & Chervin, 2014; Lund et al., 2010). In a large multi-university study, more than one-third of students reported sleeping fewer than 7 h per night, and delayed sleep onset was common (Becker et al., 2018). In addition, insomnia symptoms are frequently reported among undergraduate students, although prevalence estimates vary substantially depending on setting and measurement approaches (Gardani et al., 2022; Spyridonidis et al., 2025; H. Zhang et al., 2026).

Sleep disturbance is strongly associated with mood. A meta-analysis of longitudinal epidemiological studies has shown that insomnia predicts the onset of depression (Baglioni et al., 2011), and systematic reviews suggest a bidirectional relationship between sleep disturbance and depression symptoms (Alvaro et al., 2013; Freeman et al., 2020; Yasugaki et al., 2025). Short sleep duration has also been associated with increased risk of mental disorders, particularly anxiety and depression, although effect estimates vary by study design and sleep assessment methods (J. Zhang et al., 2024). Cross-sectional studies in university populations similarly report consistent associations between poorer sleep quality or shorter sleep and higher depressive symptom burden (Li et al., 2020).

Sleep is also relevant to positive functioning. Longitudinal evidence indicates that better sleep is associated with higher psychological well-being and fewer internalizing symptoms (Bacaro et al., 2024; O’Callaghan et al., 2021; Vestergaard et al., 2024). Randomized controlled trials further suggest that improving sleep can reduce depressive symptoms and improve broader mental health outcomes, although effect sizes and generalizability vary across interventions and populations (Scott et al., 2021). Because sleep and mental health are embedded within broader behavioral patterns, including physical activity, sedentary time, and tobacco use, adjustment for these characteristics is advisable in observational analyses, without implying that any single behavior fully accounts for observed associations (Memon et al., 2021). These distinctions suggest that individual sleep characteristics may relate differently to negative versus positive mental health dimensions: nighttime disturbance and insomnia-related features may align more with depressive symptoms, whereas restorative aspects such as adequate duration and good quality may be more relevant to positive well-being.

Evidence from Kuwait and neighboring Gulf countries indicates that sleep health is an important concern in university settings. At Kuwait University, awareness of and practices related to sleep hygiene have been associated with sleep quality (Al-Kandari et al., 2017), while local evidence has linked academic stress to poorer lifestyle behaviors, including suboptimal sleep (AlHamlan et al., 2025). Studies from Saudi university populations similarly report associations between sleep duration or quality and depressive symptoms, stress, and anxiety (Alqurashi et al., 2022; Alwhaibi & Al Aloola, 2023). A regional systematic review has also estimated a high but heterogeneous prevalence of insomnia symptoms (Baklola et al., 2024). However, much of the existing regional literature has focused on global sleep quality scores or a single sleep dimension, has primarily examined psychological distress outcomes without considering positive well-being, or has not addressed potential overlap between sleep-related content and mental health measures.

Measurement overlap is particularly relevant in studies examining sleep and mental health. The PHQ-9 includes an item assessing sleep disturbance, and the WHO-5 includes an item related to feeling rested upon waking (Kroenke et al., 2001; Topp et al., 2015; World Health Organization, 2024). Consequently, observed associations between sleep measures and these outcomes may partly reflect shared content rather than independent relationships. Related evidence from an older-adult NHANES population showed that adjustment for the sleep-related PHQ-9 item attenuated sleep–depression associations; associations with overall sleep patterns and trouble sleeping persisted, whereas the association with short sleep duration was no longer statistically significant (Niu et al., 2025). Empirical precedent supports examining this directly: a recent network analysis of depression, anxiety, and sleep problems among university students reconstructed the symptom networks after excluding PHQ-9 item 3 and reported that the overall topology and key findings remained stable (Wu & Wang, 2026). Sensitivity analyses that exclude overlapping items can help assess whether associations persist after minimizing this source of measurement dependence, while retaining the complete administered scores as primary outcomes.

Accordingly, this cross-sectional study examined associations between four brief self-reported sleep characteristics, including self-rated sleep quality, sleep disturbance frequency, sleep duration, and sleep-onset latency, and depressive symptom burden and psychological well-being among students at Kuwait University. We hypothesized that poorer sleep quality, more frequent sleep disturbance, shorter sleep duration, and longer sleep-onset latency would be associated with higher depressive-symptom burden and lower psychological well-being after adjustment for sociodemographic and lifestyle factors. Additional analyses examined whether these associations persisted after reducing direct sleep-related item overlap. We also explored whether individual sleep characteristics showed different patterns of association across depressive symptoms and positive well-being, and whether associations differed by sex. Given the cross-sectional design, findings are interpreted as associations rather than causal relationships.

## 2. Materials and Methods

### 2.1. Study Design, Setting, and Participants

**Figure 1:**
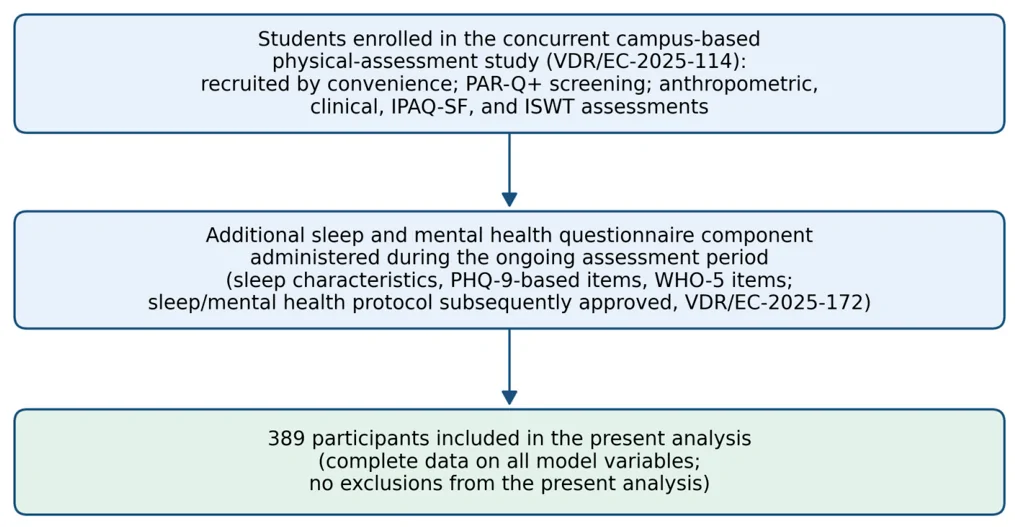
Participant flow for the present cross-sectional analysis. Participants were drawn from the concurrent campus-based physical-assessment study (VDR/EC-2025-114), which used PAR-Q+ screening. The additional sleep and mental health questionnaire component was administered during the ongoing assessment period; a separate sleep, sedentary behavior, and mental health protocol (VDR/EC-2025-172) was subsequently approved. In total, 389 participants with complete data on all model variables were included, with no exclusions from the present analysis. Counts of students unavailable because of the concurrent study’s criteria were not retained in the present analytic dataset. Height and weight used to calculate BMI were obtained from the concurrent physical-assessment study.

This cross-sectional study examined associations between sleep characteristics and mental health among Kuwait University students participating in an ongoing campus-based health assessment project. Participants were originally recruited under a separately approved physical-assessment study (VDR/EC-2025-114), which included participant recruitment, anthropometric and clinical measurements, IPAQ-SF, sedentary behavior, smoking status, and Incremental Shuttle Walk Test assessment. During the ongoing participant assessment period, additional questionnaire items addressing sleep characteristics and mental health were administered. A separate protocol addressing sleep, sedentary behavior, and mental health was subsequently approved as VDR/EC-2025-172. The present manuscript examines the sleep and mental health research question and does not analyze physical performance outcomes (e.g., shuttle distance, walking time, oxygen saturation, or perceived exertion) from the concurrent study. The questionnaire assessed sleep characteristics, depressive symptoms, well-being, physical activity and sedentary behavior, smoking/vaping, and sociodemographic characteristics, and was administered electronically by trained research assistants who read the items to participants and entered their responses into Google Forms. Height and weight used to calculate BMI were obtained from the concurrent physical-assessment study (VDR/EC-2025-114) and linked at the participant level. All participants provided electronic informed consent before participation; responses were confidential and participants could withdraw at any time. Known medical conditions were assessed using a checklist that included a mental health condition response option. No participant in the analytic sample selected this option. Mental health conditions were self-reported and were not independently clinically verified, and psychiatric conditions were not an a priori exclusion criterion. The present study included all 389 participants who had complete data for the sleep characteristics, mental health outcomes, and covariates entered in the primary regression models; no participants were excluded from the primary analysis because of missing data. The concurrent physical-assessment study recruited participants using a pragmatic convenience-sampling approach across faculties and colleges on the main Kuwait University campus in Kuwait City, through campus-based recruitment sessions, posted notices, in-class and faculty-distributed announcements, and word-of-mouth referrals. The additional questionnaire component was administered to participants enrolled in the concurrent physical-assessment study. Eligibility for the present study required current enrollment at Kuwait University, age 18 years or older, ability to understand the English-language study procedures and questionnaire, and provision of electronic informed consent. No exclusions were applied on the basis of sex, nationality, body weight, or academic year. Because participants were recruited from among students concurrently participating in a separate physical-assessment study, whose eligibility for physical testing had been assessed using the Physical Activity Readiness Questionnaire for Everyone (PAR-Q+) and related safety criteria (Warburton et al., 2011), the sampling frame may have been shaped by that study’s physical readiness requirements. Participant flow is summarized in Figure 1. The questionnaire was administered in English only; no Arabic-language versions of the PHQ-9, WHO-5, IPAQ-SF, or the brief sleep items were used, and no translation or back-translation procedure was undertaken for the present study. The sample included 297 female and 92 male students (mean age 20.2 ± 2.3 years), of whom 80.2% were Kuwaiti. The proportion of female participants (76.3%) was broadly similar to the female proportion of the enrolled Kuwait University student population (71.9%) (Kuwait University, 2025); this indicates that the sample’s sex distribution was not markedly atypical of the student body, but it does not establish that the convenience sample was representative in other respects.

Participants were recruited and the anthropometric and clinical measurements were obtained under the concurrent physical-assessment study (VDR/EC-2025-114; approved 26 May 2025). Additional questionnaire items addressing sleep characteristics and mental health were administered during the ongoing assessment period, and a separate protocol addressing sleep, sedentary behavior, and mental health was subsequently approved by the same committee (VDR/EC-2025-172). Height and weight used to calculate BMI were obtained from the concurrent physical-assessment study and linked at the participant level.

No separate a priori sample-size calculation was performed specifically for the multivariable regression analyses reported in the present manuscript. The present analysis included all 389 participants with complete data for the selected sleep characteristics, mental health outcomes, and covariates. The primary multivariable model contained 11 predictor parameters, and effect estimates are presented with 95% confidence intervals to indicate their precision; the analyses estimate adjusted associations rather than population-prevalence estimates. The sample is a single-university convenience sample rather than a representative sample of Kuwait University students.

### 2.2. Sleep Characteristics

Consistent with multidimensional models of sleep health (Buysse, 2014), four brief self-reported sleep characteristics were assessed using single survey items. Overall sleep quality was self-rated as very bad, fairly bad, fairly good, or very good and coded from 1 to 4, with higher scores indicating better quality. Sleep disturbance frequency was assessed as the frequency of sleep disruption due to discomfort or waking (never, 1–2 times per week, or 3 or more times per week). Sleep duration was recorded as the usual number of hours of sleep per night and entered as a continuous variable, and sleep-onset latency as the usual time taken to fall asleep (minutes). The brief sleep items were not separately psychometrically validated or formally pilot-tested. These four characteristics were selected to capture complementary aspects of sleep (quality, continuity, quantity, and initiation) that are amenable to brief self-report in a large field study; they do not constitute a comprehensive multidimensional sleep assessment. Other collected items (usual bedtime and wake time, and sleep-medication frequency) were not analyzed as exposures, because a single usual clock time does not index sleep regularity and sleep-medication use may reflect treatment or underlying morbidity rather than an independent dimension of sleep health.

Because these brief items do not constitute a validated multidimensional sleep instrument, such as the Pittsburgh Sleep Quality Index, findings should be interpreted accordingly. Sleep-onset latency was positively skewed (range, 1–180 min) and was therefore analyzed as the natural logarithm of (minutes + 1) in the primary analyses. Raw sleep-onset latency and a dichotomous variable (>30 min) were examined in untabulated sensitivity analyses; these did not materially change the latency findings. Sleep quality and sleep disturbance frequency were entered as ordinal variables; regression coefficients therefore represent the estimated difference associated with a one-category change.

### 2.3. Depressive Symptoms

Depressive symptoms during the previous two weeks were assessed using a nine-item PHQ-9-based depressive-symptom measure, each item scored 0–3, with total scores ranging from 0 to 27 (Kroenke et al., 2001). Two items were administered with modified wording: item 3 as “Trouble falling or staying asleep” (omitting the standard “or sleeping too much” component) and item 9 as “Thoughts of self-harm”; response anchors and the two-week timeframe were otherwise retained. For descriptive purposes only, the conventional PHQ-9 cut point of ≥10 was applied; because two item stems differed from standard PHQ-9 wording, this was not interpreted as a validated prevalence estimate or clinical diagnosis. Because the fielded PHQ-9-based item 3 assessed trouble falling or staying asleep, a modified score excluding item 3 was calculated for measurement-overlap sensitivity analyses. This modified score does not correspond to the conventional PHQ-8, which excludes item 9 (the self-harm item) rather than item 3; PHQ-8 cut-offs were therefore not applied.

### 2.4. Psychological Well-Being

Psychological well-being during the previous two weeks was measured using the five WHO-5 items, administered with modified, frequency-based response anchors (Never, Rarely, Sometimes, Often, Very often, Always; scored 0–5) rather than the standard time-based anchors. Item scores were summed (raw score 0–25) and multiplied by four to obtain a 0–100 modified WHO-5 score, which served as the primary well-being outcome; no WHO-5 clinical cut-offs or normative comparisons were applied. Because the WHO-5 includes an item on waking “fresh and rested,” a modified four-item score, excluding this item, was calculated for a measurement-overlap sensitivity analysis and reported as the mean item score (0–5). Standard WHO-5 cut-offs were not applied to the modified score. Higher WHO-5 scores indicate better well-being and were analyzed as a positive outcome rather than the inverse of depressive symptoms.

### 2.5. Covariates

Covariates were selected based on plausible associations with both sleep and mental health outcomes (Memon et al., 2021). These included age (years), sex (female/male), and nationality (Kuwaiti or non-Kuwaiti); physical activity category derived from the short-form of the International Physical Activity Questionnaire (IPAQ; low, moderate, or high) (Craig et al., 2003), with low activity as the reference; weekday sitting time (per 60 min/day); current cigarette smoking and/or vaping; and body mass index (BMI; kg/m^2^), calculated from measured height and weight. Because frequency counts were sparse and contained implausible self-reported values, current cigarette smoking and/or vaping was modeled as a binary indicator (current occasional or daily smoking and/or vaping versus neither).

### 2.6. Statistical Analysis

Associations were examined using multivariable linear regression with sequential adjustment. Model 1 adjusted for age, sex, and nationality. Model 2 (the primary model) additionally adjusted for IPAQ activity category, weekday sitting time (per 60 min/day), and current cigarette smoking and/or vaping (retaining the Model 1 covariates). Model 3 additionally adjusted for BMI as a sensitivity analysis. Because the cross-sectional design cannot establish temporal or causal ordering, BMI was not treated as a definitively confounding or mediating variable.

The four sleep characteristics were entered simultaneously into all models to estimate their mutually adjusted associations. Depressive-symptom burden (PHQ-9-based total score) was the primary outcome, and psychological well-being (modified WHO-5 score) was the secondary outcome. Unstandardized regression coefficients (B) with 95% confidence intervals and standardized coefficients (β) are reported, together with the model *R*^2^ and adjusted *R*^2^.

Sensitivity analyses repeated the depressive symptom models using the PHQ-9-based score excluding item 3, and the well-being models using the modified four-item WHO-5 score to reduce measurement overlap (Kroenke et al., 2001; Topp et al., 2015; World Health Organization, 2024). Robustness of the PHQ-9 models was further evaluated using 5000-sample bias-corrected and accelerated (BCa) bootstrap regressions, with fixed Mersenne Twister initialization values to support reproducibility.

An exploratory analysis examined a study-derived count of adverse sleep characteristics (sleep duration <7 h (Watson et al., 2015), sleep-onset latency >30 min (Ohayon et al., 2017), fairly or very bad sleep quality, and sleep disturbance at least three times per week; range 0–4). Associations were estimated using the PHQ-9-based score excluding item 3 to avoid direct overlap with the sleep-based composite.

Multicollinearity was assessed using variance inflation factors (all <1.6). Residual distributions, standardized residuals, leverage values, and Cook’s distance were examined to assess model assumptions and influential observations. Because residual normality was imperfect in the PHQ-9 models, BCa bootstrap analyses were used to provide an inferential sensitivity analysis.

Continuous variables were compared using independent samples *t*-tests, with Welch’s correction applied when Levene’s test indicated unequal variances, and categorical variables using the Pearson’s chi-square test without continuity correction (Fisher’s exact test where any expected cell count was below five). Non-normally distributed continuous variables (e.g., sleep-onset latency) were compared using the Mann–Whitney U test. The proportion of participants at or above the conventional PHQ-9 cut point is reported with a Wilson 95% confidence interval. As a sensitivity analysis, the primary regression model was refitted after excluding the single participant aged >40 years. All tests were two-sided, with effect estimates presented alongside 95% confidence intervals. Findings are interpreted as associations rather than causal effects. Statistical analyses were performed using IBM SPSS Statistics, version 31.0 (IBM Corp., Armonk, NY, USA). Generative artificial-intelligence tools were used during manuscript revision to assist with language refinement, manuscript organization, preparation of responses to peer-review comments, and checking the interpretation and presentation of statistical output; they were not used to generate, alter, or fabricate study data or to independently perform the statistical analyses, and all AI-assisted outputs were reviewed and verified by the authors.

Internal consistency of the outcome measures was quantified using Cronbach’s alpha and McDonald’s omega for the administered versions of the PHQ-9-based and modified WHO-5 scores and both item-removed scores. Because the PHQ-9-based and modified WHO-5 scores were non-normally distributed (Shapiro–Wilk *W*(389) = 0.972, *p* < 0.001 for the PHQ-9-based score; *W*(389) = 0.985, *p* < 0.001 for the modified WHO-5 score), their association was summarized using Spearman’s rank correlation. Two further sensitivity analyses were conducted. First, to assess whether the sleep–mental health associations differed by sex, the four sleep characteristics were each multiplied by sex and the resulting four interaction terms were added to Model 2 and tested jointly with an omnibus F-test; sex-stratified models were also estimated and are reported in the Supplementary Material. Second, because sleep quality and sleep disturbance frequency were entered as linearly ordered scores in the primary models, sensitivity models re-entered their categories as indicator (dummy) variables, and departure from linearity was assessed with an F-test comparing the categorical and linear specifications.

## 3. Results

### 3.1. Participant Characteristics

Participants (*N* = 389) had a mean age of 20.2 ± 2.3 years (range, 18–42 years); 297 (76.3%) were female, and 312 (80.2%) were Kuwaiti. The proportion of female participants (76.3%) was broadly similar to the female proportion of the enrolled Kuwait University student population (71.9%) (Kuwait University, 2025), indicating that the sample’s sex distribution was not markedly atypical of the student body, although this does not establish representativeness in other respects. Mean BMI was 25.4 ± 5.5 kg/m^2^, and 42 participants (10.8%) reported current cigarette smoking and/or vaping. Mean sleep duration was 7.0 ± 1.6 h/night, and median sleep-onset latency was 30 min (IQR 15–60). Sample characteristics are summarized in Table 1.

### 3.2. Depressive-Symptom Burden

For descriptive purposes, 139 of 389 students (35.7%; Wilson 95% CI, 31.1–40.6) scored at or above the conventional PHQ-9 cut point of ≥10; because the administered PHQ-9-based measure differed from standard wording for two items, this is reported as a sample description rather than a validated screening-prevalence estimate. This proportion was higher among female than male students (117/297, 39.4%, versus 22/92, 23.9%; χ^2^(1) = 7.33, *p* = 0.007). Mean PHQ-9-based scores were also higher in females than males (8.8 ± 4.8 versus 6.9 ± 3.7; Welch *t* = 3.87, *p* < 0.001). These results reflect depressive-symptom burden and do not represent clinical diagnoses of depression.

### 3.3. Sleep Characteristics and Depressive Symptoms

**Figure 2:**
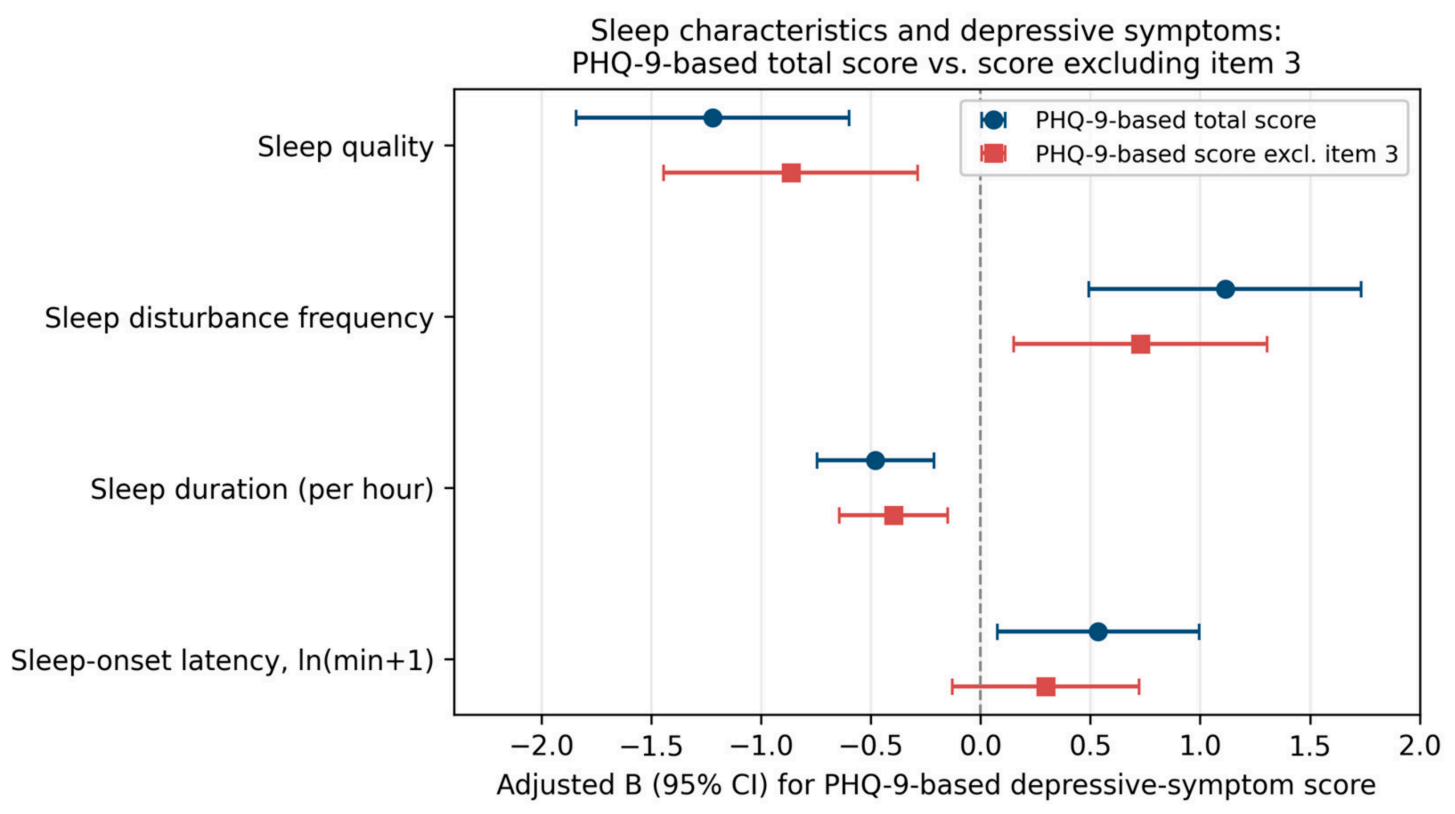
Sleep characteristics and depressive symptoms: PHQ-9-based total score vs. score excluding item 3. Points show adjusted B (95% CI) from Model 2. Item 3 is removed from the outcome score, not from the set of predictors. Because the two outcome scores have different possible ranges (0–27 and 0–24), differences in absolute B magnitude should not be interpreted as directly scale-equivalent effect-size differences.

In the main adjusted model (Model 2), poorer sleep quality, more frequent sleep disturbance, and shorter sleep duration were independently associated with higher PHQ-9-based scores, whereas sleep-onset latency showed a weaker association (Table 2; Figure 2). Each one-category increase in sleep quality was associated with a 1.22-point lower PHQ-9-based score (95% CI −1.84 to −0.60, *p* < 0.001; β = −0.20). Each step up in sleep disturbance frequency was associated with a 1.11-point higher score (0.49 to 1.73, *p* < 0.001; β = 0.18). Each additional hour of sleep was associated with a 0.48-point lower score (−0.74 to −0.21, *p* < 0.001; β = −0.17). The model explained 23.8% of the variance (adjusted *R*^2^ = 0.216). Estimates were essentially unchanged after adjustment for BMI (Model 3).

### 3.4. Sleep Characteristics and Well-Being

In analyses of WHO-5 well-being (Table 3), better sleep quality and longer sleep duration were independently associated with higher modified WHO-5 well-being scores in Model 2 (sleep quality B = 4.01, 95% CI 1.72 to 6.30, *p* < 0.001; sleep duration B = 1.35, 0.37 to 2.33, *p* = 0.007). Sleep disturbance frequency and sleep-onset latency were not independently associated. Among covariates, a high IPAQ physical activity category was independently associated with higher well-being (B = 8.92, 95% CI 4.04 to 13.81, *p* < 0.001; β = 0.19). The model explained 16.0% of the variance (adjusted *R*^2^ = 0.136).

### 3.5. Measurement-Overlap Sensitivity Analyses

When PHQ-9 item 3 was excluded, sleep quality, sleep disturbance frequency, and sleep duration remained independently and significantly associated with depressive symptom burden, whereas sleep-onset latency was no longer statistically significant (Table 4). BCa bootstrap intervals based on 5000 resamples supported this same pattern. In a modified well-being analysis excluding the “fresh and rested” item, better sleep quality and longer sleep duration remained positively and independently associated with well-being scores. These sensitivity analyses support the robustness of the main findings after reducing conceptual overlap between sleep-related predictors and outcome items.

### 3.6. Reliability, Effect Modification by Sex, and Functional-Form Sensitivity

Internal consistency (administered versions) was acceptable for both outcome measures: PHQ-9-based α = 0.73 (ω = 0.81) and modified WHO-5 α = 0.71 (ω = 0.81). The PHQ-9-based and modified WHO-5 scores were inversely correlated (Spearman ρ = −0.378, *p* < 0.001, *N* = 389). The item-removed scores used in the overlap analyses showed comparable reliability for the eight-item PHQ-9-based score (α = 0.71, ω = 0.80) and somewhat lower alpha for the four-item modified well-being score (α = 0.64, ω = 0.78), as expected given the reduced item count.

There was no evidence that the sleep–mental health associations differed by sex. Adding the four sleep-by-sex interaction terms to Model 2 did not improve model fit for either outcome (PHQ-9: *R*^2^ 0.238 to 0.241, joint *F*(4, 373) = 0.40, *p* = 0.81; WHO-5: *R*^2^ 0.160 to 0.169, joint *F*(4, 373) = 1.00, *p* = 0.41). Most sex-stratified estimates were directionally similar across female and male students, although confidence intervals were substantially wider among male students (*n* = 92); these are presented in Supplementary Table S1. These nonsignificant interactions indicate no detected effect modification by sex rather than evidence that the associations are identical in female and male students, and power to detect interaction was limited by the smaller male subgroup.

For the PHQ-9-based outcome, modeling sleep quality and sleep disturbance frequency as categorical indicators rather than linear scores did not improve fit (categorical *R*^2^ = 0.239 versus linear *R*^2^ = 0.238; departure from linearity *F*(3, 374) = 0.12, *p* = 0.95), and the category-specific coefficients were monotonic (sleep quality, relative to very bad: fairly bad B = −1.83, fairly good B = −2.90, very good B = −4.05; sleep disturbance, relative to never: 1–2 times/week B = +1.23, ≥3 times/week B = +2.29). This supports the linear ordinal specification for the depressive-symptom models. For the modified WHO-5 outcome, however, the categorical specification fitted better than the linear one (categorical *R*^2^ = 0.185 versus linear *R*^2^ = 0.160; *F*(3, 374) = 3.74, *p* = 0.01), indicating departure from linearity. The WHO-5 sleep-quality association should therefore be interpreted as an overall trend rather than a strictly uniform per-category increment, and further details of the categorical specification are reported in Supplementary Table S2.

### 3.7. Exploratory Adverse Sleep Characteristic Burden (Supplementary)

A higher count of adverse sleep characteristics, including sleep duration <7 h, sleep-onset latency >30 min, fairly or very bad sleep quality, and sleep disturbance at least 3 times per week, was associated with higher depressive symptom burden and lower well-being (Supplementary Table S3). Each additional adverse sleep characteristic was associated with a 1.09-point higher PHQ-9-based score excluding item 3 (95% CI, 0.76 to 1.41; *p* < 0.001) and a 3.09-point lower modified WHO-5 score (95% CI, −4.40 to −1.79; *p* < 0.001). Because category means were not strictly monotonic, these findings are interpreted as exploratory associations rather than a graded or dose–response relationship.

**Sensitivity check.** Excluding the single participant aged 42 years (*N* = 388) left the primary PHQ-9 Model 2 estimates essentially unchanged (quality −1.22, sleep disturbance frequency 1.11, duration −0.48, latency[ln] 0.54; *R*^2^ = 0.234).

## 4. Discussion

In this cross-sectional analysis of 389 university students at Kuwait University, multiple self-reported sleep characteristics were independently associated with mental health after adjustment for demographic and lifestyle factors. Poorer sleep quality, more frequent sleep disturbance, and shorter sleep duration were associated with higher depressive symptom burden, while better sleep quality and longer sleep duration were associated with higher psychological well-being. More than one-third of participants scored ≥10 on the PHQ-9-based measure when the conventional cut point was applied descriptively, with a higher proportion among female students. These findings extend previous evidence from Kuwait and the broader region (Al-Kandari et al., 2017; AlHamlan et al., 2025; Alqurashi et al., 2022; Alwhaibi & Al Aloola, 2023; Baklola et al., 2024) by simultaneously modeling multiple sleep dimensions and examining their associations with both negative and positive mental health outcomes.

Sleep quality, sleep disturbance frequency, and sleep duration showed consistent associations with depressive symptoms across all models, including after exclusion of PHQ-9 item 3. Bootstrap analyses supported these findings. This pattern is broadly consistent with a large NHANES-based study of older adults, in which adjustment for the sleep-related PHQ-9 item attenuated individual sleep–depression associations; associations with overall sleep patterns and trouble sleeping persisted, whereas the association with short sleep duration was no longer statistically significant (Niu et al., 2025). Although that population differs substantially from the present university sample, the methodological implication is similar: addressing overlapping sleep content can alter specific associations without necessarily eliminating the broader sleep–depression relationship. Sleep-onset latency showed weaker and less consistent associations across outcomes; this cross-outcome difference is described as exploratory rather than as a prespecified dimension-specific effect. This dissociation—whereby sleep disturbance frequency is related to depressive symptoms but not to positive well-being—is consistent with two-dimensional models of affect, in which positive and negative affects are partly separable dimensions and nighttime disturbance may act as a specific correlate of negative affect rather than of positive functioning. Moreover, because the fielded item 3 assessed difficulty falling or staying asleep, the attenuation of the latency association after excluding that item suggests that the full-score association may have been partly influenced by shared insomnia-related content. Although overlap between sleep and symptom measures cannot be fully ruled out, sensitivity analyses reduce concerns that the findings are solely driven by shared item content (Kroenke et al., 2001; Topp et al., 2015; World Health Organization, 2024). These results align with longitudinal and meta-analytic evidence linking sleep disturbance and short sleep duration with depressive symptoms and bidirectional relationships over time (Alvaro et al., 2013; Baglioni et al., 2011; Freeman et al., 2020; Yasugaki et al., 2025; J. Zhang et al., 2024).

Sleep characteristics showed differential associations with well-being and depressive symptoms. Sleep quality and duration were associated with both outcomes, whereas the frequency of sleep disturbance was associated only with depressive symptoms. This pattern aligns with multidimensional models of sleep health (Buysse, 2014) and longitudinal evidence linking sleep to positive psychological well-being and to internalizing symptoms (Bacaro et al., 2024; O’Callaghan et al., 2021; Vestergaard et al., 2024), suggesting that the restorative aspects of sleep may be more closely linked to positive psychological functioning than to the absence of disturbance.

The independent association between high self-reported physical activity and higher psychological well-being should be interpreted specifically in relation to reported activity behavior rather than as evidence of greater functional capacity. In related Kuwait University research, IPAQ-SF activity showed only weak association and limited individual-level agreement with field-assessed functional capacity, whereas field-assessed functional capacity was associated with physical activity volume and BMI (Salman, 2026a, 2026b). These findings support treating self-reported physical activity and functional capacity as related but non-interchangeable constructs.

The descriptive cut-point findings, including the higher proportion among female students, are consistent with widely reported sex differences in depressive-symptom screening among young adults and with the substantial mental health burden documented in university populations (Auerbach et al., 2018; Becker et al., 2018). However, these results reflect depressive-symptom burden rather than clinical diagnosis and should be interpreted accordingly; they are not directly comparable with prevalence estimates derived from different instruments or thresholds. Together with the sleep findings, they highlight sleep as a potentially actionable focus within broader campus mental health and well-being initiatives. While randomized evidence indicates that improving sleep can benefit mental health in some populations (Scott et al., 2021), the present cross-sectional design cannot establish that addressing sleep would reduce symptoms in this setting, and screening alone should not be assumed to improve outcomes without linked support. At Kuwait University, candidate approaches for future evaluation could include brief sleep hygiene education within student support services and assessment of whether academic scheduling practices, particularly early academic commitments, are compatible with adequate sleep duration; their effectiveness cannot be inferred from the present cross-sectional findings and requires prospective evaluation.

In the exploratory analyses, a higher count of adverse sleep characteristics was associated with greater symptom burden and lower well-being. Because the category-specific means were not perfectly monotonic, this composite is interpreted as an exploratory, adjusted association with the count of adverse sleep characteristics rather than a graded or dose–response relationship, and it is study-derived rather than a validated index. It is presented to illustrate the cumulative patterning of self-reported sleep problems and should be confirmed in independent samples.

This study has several strengths. These include the concurrent modeling of multiple sleep characteristics against both symptom burden and positive well-being outcomes, the handling of measurement overlap through item-removed scores, bootstrap confirmation with fixed seeds, adjustment for physical activity, sitting time, and current cigarette smoking and/or vaping (Memon et al., 2021), and a complete data analytic sample.

Several limitations should also be noted. The cross-sectional design precludes causal inference and cannot resolve temporal ordering or reverse causation; in particular, the role of body mass index cannot be determined. Sleep was assessed with brief single self-report items rather than a validated multidimensional instrument such as the Pittsburgh Sleep Quality Index, so the associations may be affected by reporting bias and shared-method variance, and no objective or actigraphic sleep measure was available. Self-reported sleep may also differ from objectively measured sleep duration and timing, as imperfect agreement between questionnaire-based and actigraphic estimates has been documented (Lauderdale et al., 2008). Participants were drawn from a single university by convenience and were predominantly female, which may limit generalizability. In addition, because the sample size was not derived from a formal a priori power calculation for the specific multivariable models reported here, adequate statistical power to detect small effects cannot be assured. Because participants were recruited from among students concurrently participating in a separate physical-assessment study that used physical-readiness screening, students unable or unwilling to undertake physical testing may have been underrepresented. This recruitment pathway may therefore further limit generalizability to the wider Kuwait University student population. The adverse sleep characteristic count is a study-derived composite, and residual confounding by unmeasured factors, including academic stress and physical health conditions, cannot be excluded (AlHamlan et al., 2025). Finally, the conventional cut point indicator reflects symptom burden rather than diagnosis. Future longitudinal studies using validated and objective sleep measures, and ideally incorporating intervention components, are needed to clarify the direction and mechanisms of these associations (Bacaro et al., 2024; Scott et al., 2021; Vestergaard et al., 2024).

In addition, the depressive-symptom measure was a PHQ-9-based measure with modified wording for items 3 and 9, the well-being measure was a modified WHO-5 with frequency-based response anchors, and the brief sleep characteristic items were not part of a validated multidimensional sleep instrument; scores are therefore not directly comparable with published norms or cut-offs. The questionnaire was administered in English only and was read to participants by trained research assistants, who entered the responses; this interviewer-administered format may have introduced social desirability bias, particularly for sensitive items such as those concerning self-harm and smoking or vaping, and it may also have limited participation among students less comfortable with English. Finally, the sample was recruited by convenience from a single university, and although its sex distribution was broadly similar to that of the enrolled student population, this does not establish representativeness.

## 5. Conclusions

Among students at Kuwait University, poorer self-rated sleep quality, more frequent sleep disturbance, and shorter sleep duration were independently associated with greater depressive symptom burden, while better sleep quality and longer sleep duration were independently associated with higher psychological well-being. These associations remained robust in sensitivity analyses addressing item overlap, although sleep-onset latency was less consistent.

In this convenience sample, 35.7% scored ≥10 on the PHQ-9-based measure when the conventional cut point was applied descriptively, with a higher proportion among female students. While findings are associative and do not imply causality, they highlight sleep as a potentially modifiable target within student mental health and well-being initiatives. The study-derived adverse sleep characteristic count should be regarded as exploratory and not as a validated index or dose–response measure. Longitudinal and intervention studies are needed to clarify temporal relationships and potential causal pathways; prospective designs combining objective actigraphy with ecological momentary assessment and validated Arabic-language instruments would be particularly informative. More broadly, these findings underscore the value of routinely applying item-overlap sensitivity analyses when behavioral exposures and psychological distress measures share content, so that observed associations are not mistaken for measurement artifacts.

## Figures and Tables

**Table 1:** Participant characteristics, overall and by sex (*N* = 389).

Characteristic	Overall (*N* = 389)	Female (*n* = 297)	Male (*n* = 92)	*p*
Age, years—mean ± *SD*	20.2 ± 2.3	20.0 ± 2.2	20.8 ± 2.6	0.006
Kuwaiti nationality—*n* (%)	312 (80.2)	237 (79.8)	75 (81.5)	0.717
Body mass index, kg/m^2^—mean ± *SD*	25.4 ± 5.5	24.8 ± 5.0	27.3 ± 6.5	0.001
Current cigarette smoking and/or vaping—*n* (%)	42 (10.8)	7 (2.4)	35 (38.0)	<0.001
IPAQ category: low/moderate/high—*n* (%)	205/128/56	180/94/23	25/34/33	<0.001
Sitting time, min/day—mean ± *SD*	289 ± 133	284 ± 134	308 ± 130	0.122
Sleep duration, hours—mean ± *SD*	6.97 ± 1.62	7.06 ± 1.72	6.66 ± 1.22	0.015
Sleep-onset latency, min—median (IQR)	30 (15–60)	30 (15–60)	30 (10–60)	0.064
Sleep quality (1–4)—mean ± *SD*	2.97 ± 0.76	2.96 ± 0.76	3.02 ± 0.77	0.471
Sleep disturbance frequency (1–3)—mean ± *SD*	2.01 ± 0.76	2.09 ± 0.74	1.77 ± 0.77	<0.001
PHQ-9-based total score—mean ± *SD*	8.3 ± 4.6	8.8 ± 4.8	6.9 ± 3.7	<0.001
PHQ-9-based score ≥ 10 (conventional cut point; descriptive only)—*n* (%)	139 (35.7)	117 (39.4)	22 (23.9)	0.007
Modified WHO-5 (0–100)—mean ± *SD*	56.1 ± 16.2	55.1 ± 16.2	59.4 ± 15.9	0.028

Note. Values are mean ± *SD*, median (IQR), or *n* (%). Sex comparisons are descriptive and unadjusted: Pearson’s chi-square (without continuity correction) for categorical variables, independent samples *t*-tests (Welch where variances were unequal) for approximately normal continuous variables, and Mann–Whitney U for sleep-onset latency (median 30 min; range 1–180). The PHQ-9-based measure used modified wording for items 3 and 9, and the modified WHO-5 used frequency-based response anchors. Sleep characteristics are brief self-reported items, not a validated multidimensional sleep instrument. IQR, interquartile range; IPAQ, International Physical Activity Questionnaire; PHQ-9, Patient Health Questionnaire-9; WHO-5, World Health Organization-Five Well-Being Index.

**Table 2:** Associations between sleep characteristics and PHQ-9-based depressive-symptom scores (multivariable linear regression, *N* = 389).

Predictor	Model 1	Model 2 (Main)	Model 3 (+BMI)
Sleep quality (higher = better)	−1.24 (−1.85, −0.62) ***	−1.22 (−1.84, −0.60) ***; β = −0.20	−1.22 (−1.85, −0.60) ***
Sleep disturbance frequency (higher = more frequent)	1.08 (0.47, 1.70) ***	1.11 (0.49, 1.73) ***; β = 0.18	1.11 (0.49, 1.73) ***
Sleep duration (per hour)	−0.48 (−0.75, −0.22) ***	−0.48 (−0.74, −0.21) ***; β = −0.17	−0.47 (−0.74, −0.21) ***
Sleep-onset latency, ln(min + 1)	0.56 (0.10, 1.01) *	0.54 (0.08, 0.99) *; β = 0.11	0.55 (0.09, 1.01) *
Age (years)	−0.18 (−0.37, −0.00) *	−0.18 (−0.36, 0.01)	−0.18 (−0.37, 0.00)
Male (ref. female)	−1.33 (−2.33, −0.34) **	−1.64 (−2.83, −0.44) **	−1.69 (−2.89, −0.48) **
Non-Kuwaiti (ref. Kuwaiti)	0.07 (−0.99, 1.13)	0.06 (−1.00, 1.13)	0.07 (−1.00, 1.13)
IPAQ moderate (ref. low)	—	−0.24 (−1.17, 0.70)	−0.27 (−1.21, 0.67)
IPAQ high (ref. low)	—	0.25 (−1.07, 1.58)	0.27 (−1.06, 1.60)
Sitting (per 60 min/day)	—	0.11 (−0.08, 0.30)	0.11 (−0.07, 0.30)
Current cigarette smoking and/or vaping	—	0.56 (−0.96, 2.08)	0.52 (−1.01, 2.05)
Body mass index	—	—	0.03 (−0.05, 0.10)
**Model fit, *R*^2^; adjusted *R*^2^**	*R*^2^ 0.233; adj 0.219	*R*^2^ 0.238; adj 0.216	*R*^2^ 0.239; adj 0.215

Note. Cells show unstandardized B (95% CI). * *p* < 0.05, ** *p* < 0.01, *** *p* < 0.001. Reference groups: female, Kuwaiti, IPAQ low. Standardized β (Model 2): quality −0.20, sleep disturbance frequency 0.18, duration −0.17, latency 0.11. —, not entered. B, unstandardized regression coefficient; CI, confidence interval; ln, natural logarithm; BMI, body mass index; IPAQ, International Physical Activity Questionnaire; PHQ-9, Patient Health Questionnaire-9.

**Table 3:** Associations between sleep characteristics and modified WHO-5 well-being scores (multivariable linear regression, *N* = 389).

Predictor	Model 1	Model 2 (Main)	Model 3 (+BMI)
Sleep quality (higher = better)	3.73 (1.41, 6.04) **	4.01 (1.72, 6.30) ***; β = 0.19	4.03 (1.73, 6.32) ***
Sleep disturbance frequency (higher = more frequent)	−1.54 (−3.85, 0.77)	−1.21 (−3.50, 1.07); β = −0.06	−1.20 (−3.48, 1.09)
Sleep duration (per hour)	1.44 (0.44, 2.44) **	1.35 (0.37, 2.33) **; β = 0.14	1.32 (0.34, 2.31) **
Sleep-onset latency, ln(min + 1)	−0.58 (−2.29, 1.13)	−0.90 (−2.59, 0.79); β = −0.05	−0.97 (−2.67, 0.73)
Age (years)	0.79 (0.10, 1.48) *	0.78 (0.10, 1.46) *	0.80 (0.12, 1.48) *
Male (ref. female)	3.26 (−0.49, 7.00)	0.09 (−4.32, 4.49)	0.35 (−4.09, 4.78)
Non-Kuwaiti (ref. Kuwaiti)	−3.44 (−7.44, 0.55)	−3.76 (−7.69, 0.17)	−3.79 (−7.72, 0.14)
IPAQ moderate (ref. low)	—	3.60 (0.15, 7.05) *	3.76 (0.30, 7.23) *
IPAQ high (ref. low)	—	8.92 (4.04, 13.81) ***	8.84 (3.95, 13.72) ***
Sitting (per 60 min/day)	—	−0.67 (−1.36, 0.02)	−0.68 (−1.37, 0.01)
Current cigarette smoking and/or vaping	—	2.03 (−3.58, 7.64)	2.22 (−3.41, 7.84)
Body mass index	—	—	−0.14 (−0.43, 0.14)
**Model fit, *R*^2^; adjusted *R*^2^**	*R*^2^ 0.115; adj 0.099	*R*^2^ 0.160; adj 0.136	*R*^2^ 0.162; adj 0.136

Note. Cells show unstandardized B (95% CI). * *p* < 0.05, ** *p* < 0.01, *** *p* < 0.001. Reference groups: female, Kuwaiti, IPAQ low. Standardized β (Model 2): quality 0.19, duration 0.14, sleep disturbance frequency −0.06, latency −0.05. —, not entered. B, unstandardized regression coefficient; CI, confidence interval; ln, natural logarithm; BMI, body mass index; IPAQ, International Physical Activity Questionnaire; WHO-5, World Health Organization-Five Well-Being Index.

**Table 4:** Measurement-overlap sensitivity analyses (Model 2, *N* = 389).

Sleep Exposure	*B* (95% CI)	β	*p*	BCa 95% CI
**PHQ-9-based total score (primary outcome)**				
Sleep quality	−1.22 (−1.84, −0.60)	−0.202	<0.001	(−1.92, −0.52)
Sleep disturbance frequency	1.11 (0.49, 1.73)	0.183	<0.001	(0.47, 1.74)
Sleep duration	−0.48 (−0.74, −0.21)	−0.169	<0.001	(−0.77, −0.19)
Sleep-onset latency (ln)	0.54 (0.08, 0.99)	0.113	0.022	(0.03, 1.03)
**PHQ-9-based score excluding item 3 (overlap sensitivity)**				
Sleep quality	−0.86 (−1.44, −0.29)	−0.161	0.003	(−1.55, −0.21)
Sleep disturbance frequency	0.73 (0.15, 1.30)	0.135	0.013	(0.13, 1.35)
Sleep duration	−0.40 (−0.65, −0.15)	−0.158	0.002	(−0.66, −0.15)
Sleep-onset latency (ln)	0.30 (−0.13, 0.72)	0.070	0.173	(−0.14, 0.73)
**Four-item well-being, mean 0–5 (excluding rested item)**				
Sleep quality	0.17 (0.06, 0.29)	0.162	0.004	—
Sleep disturbance frequency	−0.06 (−0.17, 0.06)	−0.052	0.343	—
Sleep duration	0.06 (0.01, 0.11)	0.116	0.023	—
Sleep-onset latency (ln)	−0.04 (−0.13, 0.04)	−0.050	0.338	—

Note. All models adjusted as in Model 2 (age, sex, nationality, IPAQ category, sitting time, current cigarette smoking and/or vaping). BCa 95% CI from 5000 bootstrap samples with fixed Mersenne Twister seeds; computed for the PHQ-9 outcomes only. *R*^2^: PHQ-9 0.238; PHQ-9 excl. item 3 0.165; four-item well-being 0.138. B, unstandardized regression coefficient; CI, confidence interval; BCa, bias-corrected and accelerated; ln, natural logarithm; PHQ-9, Patient Health Questionnaire-9; WHO-5, World Health Organization-Five Well-Being Index.

## Data Availability

The data presented in this study are available on request from the corresponding author due to ethical and privacy restrictions.

## Supplementary Materials

The following supporting information can be downloaded at: https://www.mdpi.com/article/10.3390/bs16081446/s1: Table S1: Sex-stratified associations of sleep characteristics with mental-health outcomes (Model 2); Table S2: Categorical (indicator-variable) specification of ordinal sleep exposures; Table S3: Exploratory adverse sleep-characteristic burden and mental-health outcomes; Figure S1: Correlations among self-reported sleep dimensions (Spearman).
