# Supplementary material for: Sleep Characteristics and Mental Health Among Kuwait University Students: Sensitivity Analyses for Item Overlap
Source: Behav Sci (Basel). 2026 Aug 21;16(8):1446. doi: 10.3390/bs16081446 (PMC13509799; doi:10.3390/bs16081446)
Supplement: Supplementary file 1 [file behavsci-16-01446-s001.zip › behavsci-4466528-supplementary.pdf]

## Supplementary Material

*Sleep Characteristics and Mental Health Among Kuwait University Students: Sensitivity Analyses for Item Overlap*

### Table S1. Sex-stratified associations of sleep characteristics with mental-health outcomes (Model 2).

| Outcome / Predictor | Female (n = 297) B (95% CI) | Male (n = 92) B (95% CI) |
|---|---|---|
| PHQ-9-based score | | |
| Sleep quality | −1.27 (−2.02, −0.52) | −1.17 (−2.28, −0.06) |
| Sleep disturbance frequency | 1.11 (0.34, 1.87) | 0.57 (−0.54, 1.67) |
| Sleep duration (per hour) | −0.54 (−0.85, −0.24) | 0.06 (−0.57, 0.68) |
| Sleep-onset latency, ln(min+1) | 0.56 (0.01, 1.11) | 0.38 (−0.43, 1.20) |
| Modified WHO-5 score | | |
| Sleep quality | 3.82 (1.16, 6.49) | 5.06 (0.04, 10.08) |
| Sleep disturbance frequency | −1.26 (−3.98, 1.46) | 0.21 (−4.79, 5.20) |
| Sleep duration (per hour) | 1.44 (0.37, 2.50) | 1.44 (−1.39, 4.27) |
| Sleep-onset latency, ln(min+1) | −0.18 (−2.12, 1.77) | −3.31 (−7.00, 0.38) |

*Each model adjusts for the covariates in Model 2 (age, nationality, IPAQ category, sitting time, current smoking/vaping), estimated separately within each sex. Sleep-by-sex interaction terms were not statistically significant in the pooled models (Section 3.6).*

### Table S2. Categorical (indicator-variable) specification of ordinal sleep exposures.

For the PHQ-9-based outcome, modelling sleep quality and sleep disturbance frequency as categorical indicators rather than linear-ordinal terms did not improve model fit ($F(3, 374) = 0.12$, $p = 0.95$), supporting the linear-ordinal specification. For the modified WHO-5 outcome, the categorical specification fitted better than the linear one ($F(3, 374) = 3.74$, $p = 0.01$), indicating departure from the joint linear-ordinal specification. In an exploratory post-hoc decomposition, this departure was attributable to sleep quality ($F(2, 375) = 5.42$, $p = 0.005$) rather than sleep disturbance frequency ($F = 0.05$, $p = 0.82$). This decomposition is exploratory and is not interpreted as a formal threshold effect.

### Table S3. Exploratory adverse sleep-characteristic burden and mental-health outcomes.

| Outcome | B per additional adverse characteristic (95% CI) | p |
|---|---|---|
| PHQ-9-based score excluding item 3 | 1.09 (0.76, 1.41) | <0.001 |
| Modified WHO-5 score | −3.09 (−4.40, −1.79) | <0.001 |

*The adverse-sleep-characteristic count (range 0–4) comprised sleep duration <7 hours, sleep-onset latency >30 minutes, fairly or very bad sleep quality, and sleep disturbance ≥3 times per week. Because category-specific means were not monotonic, this study-derived composite is exploratory and is not interpreted as a graded or dose–response relationship.*

**Figure S1. Correlations among self-reported sleep dimensions (Spearman).**

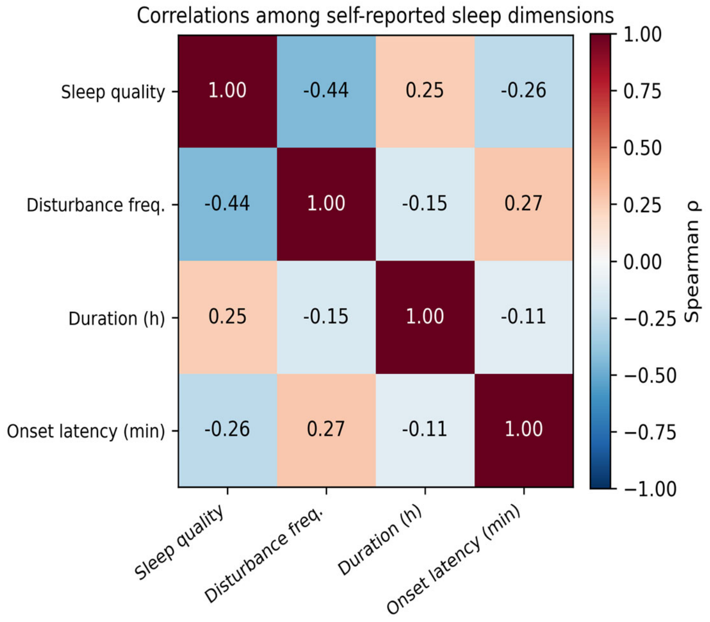


*Spearman rank correlations among the four self-reported sleep dimensions (N = 389). Inter-correlations were modest (|ρ| ≤ 0.44), supporting their treatment as distinct exposures.*
